# A Small Molecule Inhibitor Selectively Induces Apoptosis in Cells Transformed by High Risk Human Papilloma Viruses

**DOI:** 10.1371/journal.pone.0155909

**Published:** 2016-06-09

**Authors:** Amy K. Sheaffer, Min S. Lee, Huilin Qi, Susan Chaniewski, Xiaofan Zheng, Glen A. Farr, Kim Esposito, David Harden, Ming Lei, Liang Schweizer, Jacques Friborg, Michele Agler, Fiona McPhee, Robert Gentles, Brett R. Beno, Lou Chupak, Stephen Mason

**Affiliations:** Bristol-Myers Squibb, Research and Development, Wallingford, CT, United States of America; University of Nebraska-Lincoln, UNITED STATES

## Abstract

A phenotypic high-throughput cell culture screen was performed to identify compounds that prevented proliferation of the human Papilloma virus type 16 (HPV-16) transformed cell line Ca Ski. A series of quinoxaline compounds exemplified by Compound 1 was identified. Testing against a panel of cell lines demonstrated that Compound 1 selectively inhibited replication of all HPV-16, HPV-18, and HPV-31 transformed cell lines tested with 50% Inhibitory Concentration (IC_50_) values of 2 to 8 μM relative to IC_50_ values of 28 to 73 μM in HPV-negative cell lines. Treatment with Compound 1 resulted in a cascade of multiple apoptotic events, including selective activation of effector caspases 3 and 7, fragmentation of cellular DNA, and PARP (poly(ADP-ribose) polymerase) cleavage in HPV-positive cells relative to HPV-negative cells. Unregulated proliferation of HPV transformed cells is dependent on the viral oncogenes, E6 and E7. Treatment with Compound 1 resulted in a decrease in HPV E7 protein in Ca Ski cells. However, the timing of this reduction relative to other effects of compound treatment suggests that this was a consequence, rather than a cause, of the apoptotic cascade. Likewise, compound treatment resulted in no obvious effects on the E6- and E7- mediated down regulation of p53 and Rb, or their downstream effectors, p21 or PCNA. Further investigation of apoptotic signals induced by Compound 1 revealed cleavage of Caspase-8 in HPV-positive cells as early as 2 hours post-treatment, suggesting the compound initiates apoptosis through the extrinsic, death receptor-mediated, pathway of cell death. These studies provide proof of concept that cells transformed by oncogenic Papillomaviruses can be selectively induced to undergo apoptosis by compound treatment.

## Introduction

Cervical cancer is the second leading cause of cancer-related death in women ages 15–44 worldwide, and has been linked to the presence of transforming or *high risk* types of human Papilloma viruses (HPVs) [1–3]. More than 70% of cervical cancers are associated with the high risk genotypes HPV-16 and HPV-18, with less prevalent genotypes, including HPV-31, -33, -45, and -58, and together accounting for nearly all the remaining cases [1]. During the initial stages of infection, the HPV genome replicates as an episome, physically separate from the host cell genome. Replication of the episome requires a complex of two viral proteins, E1 and E2. The E1 protein acts as a helicase to unwind the viral dsDNA, while the E2 protein serves to recognize the HPV origin of replication and recruit the cellular polymerase machinery to replicate the viral genome [4, 5]. While the majority of HPV infections are believed to clear spontaneously, in the longer term, low level persistence of virus infection may result in integration of the HPV genome into the host cell and subsequent transformation of the host cell by HPV oncoproteins [6].

Integration of the HPV genome into the host cell genome coincides with an up-regulation in expression of two viral oncogenes, E6 and E7, required for cellular transformation and for ongoing replication of HPV transformed cells [7, 8]. Although E6 and E7 have been associated with the disruption of a number of cellular processes, their best-characterized functions center around maintaining cell proliferation and avoiding cell death. The E7 protein associates with cellular Rb protein targeting it for Ubiquitin-dependent degradation, thus freeing Rb-interacting protein E2F for transactivation of genes essential for the transition from G1 to S phase of the cell cycle, including the cellular DNA polymerase processivity factor PCNA (proliferating cell nuclear antigen) [9, 10]. The action of E7 alone can result in uncontrolled cell proliferation and DNA damage. Such damage would normally be sensed by cellular protein p53 resulting in a block at the G2 to M transition of the cell cycle [11]. However, in the presence of E6 protein and its co-factor, the cellular protein E6AP, p53 is also targeted for ubiquitin-dependent degradation [12, 13]. In this way, E6 and E7 work in concert to allow the cell to undergo unregulated proliferation [14]. A third HPV protein, E5, has been implicated in enhancing the transformation of cells by HPV [15, 16].

Treatment of HPV neoplasias and cancers represents a large unmet medical need. The marketed vaccines Cervarix™ and Gardasil™ have proven effective in preventing new cases of HPV infection, and hold promise for reduction in the rates of cervical cancer in the future (reviewed in [17]). However, these vaccines are not effective as therapeutics for the treatment of cervical cancer. Additionally, poor uptake of the vaccines in some markets, such as the US, means that high risk HPV infections, and resulting cervical cancers, will continue to be a concern. Recently, strategies to inactivate E6 and E7 using TALEN or CRISPR-mediated gene disruption have shown promise *in vitro* and in mouse models, but they are as yet untested in human trials [18–20]. Small molecule inhibitors of the HPV E1/E2 complex have been identified, but most are highly HPV type specific and target low risk HPV types [21, 22]. HPV E1 and E2 are no longer required to maintain cellular transformation after integration, and therefore these potential treatments would not be useful in later stages of neoplasia [7]. When high grade HPV neoplasias are identified in the cervix, current standard of care involves surgical removal of the lesion through a loop electrosurgical excision procedure (LEEP) [23]. The LEEP procedure is invasive and is not without unwanted side effects, including potential weakening of the cervix which may affect a woman’s ability to carry future pregnancies to term [24]. There are currently no FDA approved small molecule inhibitors specific for HPV. Identification of an inhibitor that could selectively kill HPV-transformed cells would therefore be of great benefit in the treatment of high grade HPV lesions and cervical cancer.

As a result of a high-throughput screening effort, we have identified a quinoxaline compound, Compound 1, which selectively inhibits the proliferation of HPV-transformed cell lines relative to HPV-negative cell lines. Our studies show that the compound does not act through inhibition of E6- and E7-mediated degradation of cellular proteins p53 and Rb. Instead, we show that proliferation is inhibited through the selective induction of the extrinsic, death receptor mediated apoptosis pathway in HPV transformed cells.

## Materials and Methods

### Cell Culture

Ca Ski, SiHa, C33A, HCT-116, and HeLa cells were obtained from the American Type Culture Collection (ATCC). J2-3T3 cells were obtained from Howard Greene (Harvard Medical School, Boston, MA) [25]. HaCaT cells were obtained from the German Cancer Research Center (Heidelberg, Germany). Ca Ski, SiHa, HeLa, C33A, Saos-2, and HaCaT were cultured in Dulbecco's Modified Eagle Medium (DMEM) supplemented with 10% fetal bovine serum (FBS). HCT-116 was cultured in Roswell Park Memorial Institute-1640 Medium (Invitrogen catalog no. 11875–093) supplemented with 10% FBS. All cells were incubated at 37°C in a humidified atmosphere with 5% CO_2_ in air, except J2-3T3 (10% CO_2_). J2-3T3 cells were cultured in DMEM containing 10% bovine calf serum. To establish the SCHPV-18 cell line, one million primary neonatal human foreskin keratinocytes (Invitrogen, catalog no. C-001-5C), cultured in KBM Gold-keratinocyte cell basal medium (Lonza), were electroporated with 5μg HPV-18 plasmid DNA (ATCC catalog no. 45152D) using the Amaxa Nucleofector II. Cells were co-transfected with 2 μg plasmid pZSGreen1-N1 (Clontech) encoding G418 resistance, and selected for four days in 100 μg/ml G418, after which selection was removed. Cells were maintained in culture six months prior to compound testing. Control cells transfected with pZSGreen1-N1 alone became senescent four days after selection and could not be expanded. To generate SCHPV-31 cells, 9E/HPV31 cells containing episomal HPV31, obtained from Craig Meyers, (Pennsylvania State University) [26] were cultured in E medium [27] in the absence of feeder cells for >3 months to induce HPV integration. Integration of the HPV-18 and HPV-31 genomes was confirmed by Southern blot (S1 Fig). SCHPV-18 and SCHPV-31 cells were maintained in E medium.

### Western Blotting

For Western blotting, cells were cultured in 6-well plates and treated the next day with compounds in a final concentration of 0.5% DMSO. Cells were lysed in RIPA buffer prior to Western blotting for p53, p21, Caspase-8, Caspase-9, PUMA, PCNA and actin, lysates were separated on NuPAGE Novex 4–12% Bis-Tris gels (Life Technologies) run in MOPS SDS Running Buffer (Life Technologies); MES Buffer (Life Technologies) was used for separation of HPV E7 proteins. NuPAGE Novex 3–8% Tris-Acetate gels (Life Tehcnologies) were used for separation of Rb and PARP proteins. The protein gels were then transferred to PVDF membranes (Life Technologies Catalog no. LC2005) in NuPage Transfer Buffer with 10% methanol. Antibodies against p53 (DO-1; used at 1:1,000 dilution), p21 (F-5; 1:200), HPV-16 E7 (NM2; 1:1000), HPV-18 E7 (F-7; 1:1,000), and actin (I-19; 1:3000) were purchased from Santa Cruz Biotechnology. Antibody against pRb was obtained from BD Bioscience (Catalog no. 554164; 1:1,000). Antibodies against Caspase-8 (1C12; 1:1,000), Caspase-9 (catalog no. 9502; 1:1,000), BID (catalog no. 2002; 1:1,000), PUMA (catalog no. 4976; 1:1000), PCNA (PC10; 1:5,000), and PARP (catalog no. 9542; 1:1000) were purchased from Cell Signaling Technology. Horseradish peroxidase conjugated secondary antibodies were purchased from Jackson ImmunoResearch Laboratories (donkey anti-mouse IgG (catalog no. 715-036-151; 1:20,000), donkey anti-rabbit IgG (Catalog no. 711-036-152; 1:20,000), donkey anti-goat IgG (catalog no. 705-035-147; 1:20,000). WesternBreeze Blocker/Diluent and Wash Solution (Life Technologies) were used for membrane blocking and washing as per manufacturer’s instructions. Chemiluminescence was detected using Amersham ECL Prime Western Blotting Detection Reagent (GE Healthcare) and exposure to Kodak BioMax MR Film.

### Cell Viability Testing

For proliferation assays, cells were plated in 96-well plates in appropriate growth medium without antiobiotics. Two hours after plating, a 200x solution of compound diluted in DMSO was added to a final concentration of 0.5% DMSO. Plates were incubated 4 days and viability was assessed using either CellTiter Blue or CellTiter Glo reagents (Promega). Data was collected using the SpectraMax Gemini EM (Molecular Devices) or the Envision Multilabel Reader (Perkin Elmer). Percent inhibition curves were fit using a four-parameter logistic formula [y = A+((B-A)/(1+((C/x)^D)))], where A and B denote minimal and maximal % inhibition, respectively, C is the IC_50_, D is the Hill slope and x represents compound concentration. IC_50_ values represent half the maximal inhibition value obtained in wells lacking viable cells.

For colony formation assays, cells were plated in 6-well plates in the absence of antibiotics in appropriate growth medium. The next day, compound diluted in DMSO was added, to a final concentration of 0.5% DMSO. Medium was replaced every 3 to 4 days with fresh medium containing compound/DMSO. On day 14, the cells were fixed by addition of neutral buffered formalin (Harleco) to 3% final concentration, then stained with 0.6% crystal violet (Sigma) dissolved in 10% ethanol, and rinsed with water before drying.

### Apoptosis Assays

For caspase-3/7 activity assays, cells were plated in 96-well plates in DMEM with 10% FBS two hours before addition of compounds in a final concentration of 0.5% DMSO, then the Caspase-Glo 3/7 Assay (Promega) was performed per manufacturer’s instructions. Luminescence was measured using the EnVision Multilabel Reader.

For flow cytometric analysis, cells were stained with Annexin V and 7-AAD using the Nexin Assay (Guava) per manufacturer’s instructions and assayed on the Guava EasyCyte. For TUNEL assays, cells were plated one day ahead onto Nunc LabTekII chamber slides (C33a cells) or 96-well imaging plates (Ca Ski cells, Becton-Dickson), then treated with compounds in a final concentration of 0.5% DMSO for 6 hours, and fixed by addition of 4% final concentration formaldehyde. Cells were permeabilized by treatment with 0.05% Triton X-100 in Dulbecco’s Phosphate Buffered Saline (DPBS) solution for 15 minutes at room temperature, then stained with EdUTP and AlexaFluor488 Azide using the Click-it® TUNEL kit (Invitrogen) as directed by the manufacturer. Cells were counter-stained with 2 μg/ml Hoechst 33342 diluted in DPBS for 15 minutes at room temperature prior to analysis. Samples were imaged using a Cellomics ArrayScan 3.5 High-content reader with a 10X objective. Images were analyzed using Compartmental Analysis v3 software. Objects were selected using nuclear fluorescence (Hoechst channel). Average pixel intensity was measured in the TUNEL stained channel and population statistics collected for objects in each well. Images were exported and processed using Adobe Photoshop software; image adjustments were performed identically across samples.

## Results

**Fig 1:**
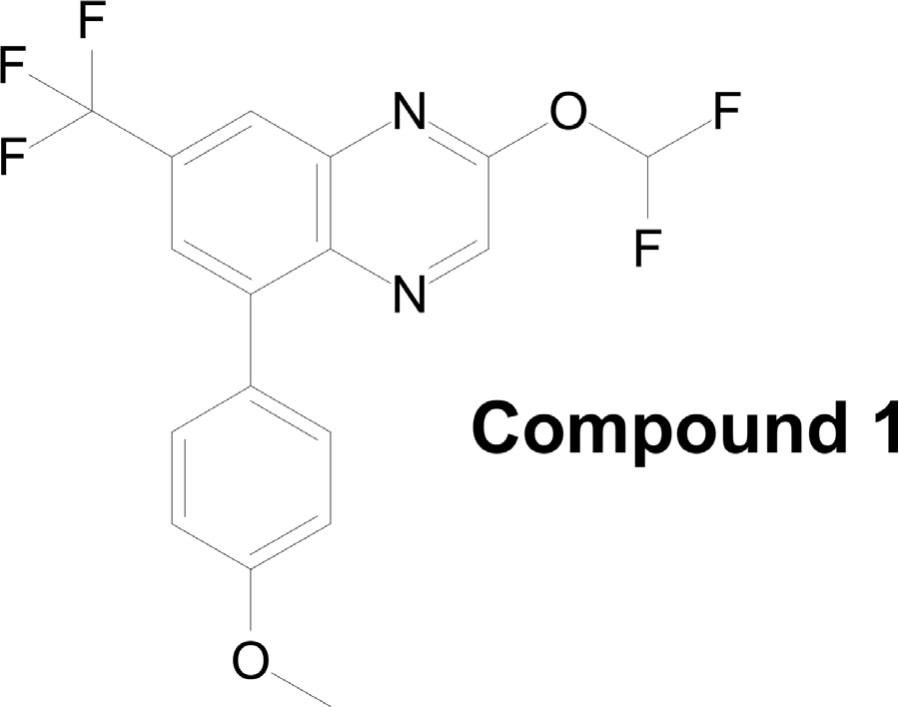
Chemical structure of the quinoxaline Compound 1. The activity of Compound 1 was tested in dose-response assays against a broader panel of cell lines, including both HPV transformed cells (Ca Ski, SiHa, HeLa, SCHPV-18, SCHPV-31, Fig 2A) and HPV-negative cells (C33a, HaCat, Saos-2, HCT-116, J2-3T3, Fig 2B).

A phenotypic high-throughput screen was designed to identify potential inhibitors of proliferation of the HPV-16 transformed Ca Ski cell line. Several hits were found through screening of this assay against the Bristol-Myers Squibb compound collection. Active compounds were further tested for activity against additional HPV-16 (SiHa) and HPV-18 (HeLa) transformed cell lines, as well as HPV-negative cell lines (Saos-2, C33a). Based upon one of the screen hits, related compounds with structural modifications were synthesized and tested for activity, resulting in a more potent and selective inhibitor, Compound 1 (Fig 1).

**Fig 2:**
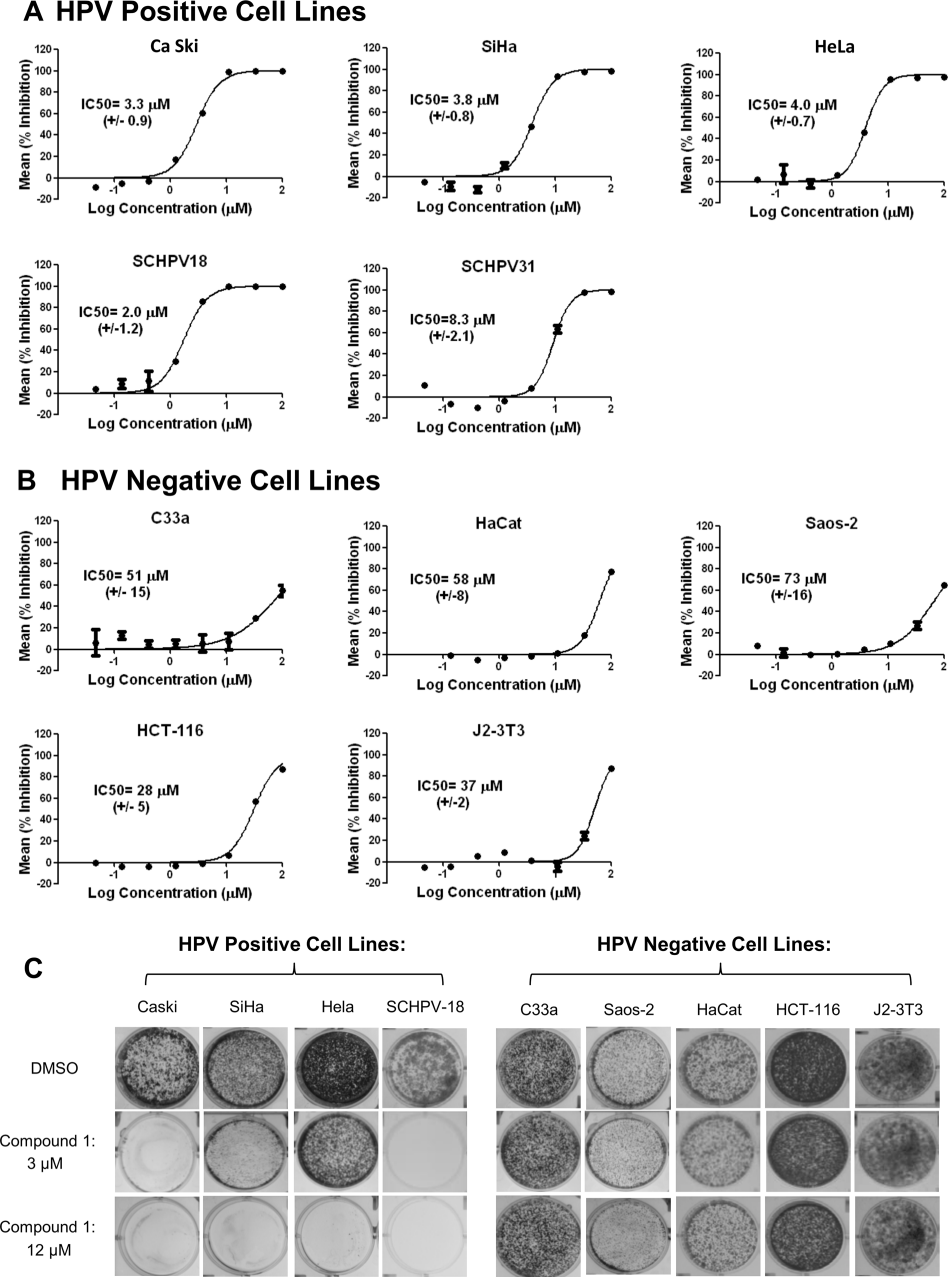
Susceptibility of HPV-positive and -negative cell lines to Compound 1. Dose response curves for HPV transformed cell lines (A) or HPV-negative cell lines (B) treated with Compound 1. The y axes depict percent inhibition of cell proliferation in viability assays (see Materials and Methods). Graphs depict a typical experiment with duplicates. IC_50_ values represent the average of ≥3 independently determined values, with standard deviation of these values in paretheses. (C) Colonies formed by the indicated HPV-positive or HPV-negative cell lines in the absence (DMSO) or presence of 3 or 12 μM Compound 1.

Cells were initially plated at a low density, then incubated for 4 days in the presence of compound dilutions to test effects on cell proliferation and viability. Effects of Compound 1 on the proliferation of HPV-transformed cells were observed at lower concentrations than those required to affect proliferation of the HPV-negative cell lines. Dose-response curves from multiple experiments were used to calculate the average concentration required to inhibit proliferation by 50%, the IC_50_ value (Fig 2A and 2B), which ranged from 2 to 4 μM on HPV-16 and HPV-18-positive cell lines to 8 μM on HPV-31-positive cells. In comparison to results with the HPV-positive cell lines, higher IC_50_ values ranging from 28 to 73 μM, were obtained on HPV-negative cell lines. The potential for selective inhibition of HPV-transformed cell proliferation by Compound 1 was confirmed in a 14-day assay for colony formation (Fig 2C). In this assay, treatment with 3 μM Compound 1 resulted in no measurable colony formation (Ca Ski and SCHPV-18 cells) or fewer colonies (SiHa and HeLa cells) than DMSO-treated controls, and treatment with 12 μM was sufficient to prevent colony formation by all 4 HPV-transformed cell lines tested. All of the HPV-negative cell lines were able to form colonies at densities similar to DMSO-treated controls after incubation with either 3 μM or 12 μM Compound 1. Taken together, these results suggest that Compound 1 is a selective inhibitor of HPV-transformed cell proliferation.

In Fig 2A, maximal inhibition of proliferation was observed at concentrations ≥ 11 μM at both days 4 and 14 using Ca ski cells. In subsequent mechanism of action experiments, those typically done with less sensitive assays for shorter periods of time, higher concentrations of Compound 1 were used in order to ensure a uniform and maximal effect. We observed that removal of compound 1 and washing the cells as early as 6 hours post-treatment resulted in a no changes in the IC50 value obtained in a 4 day proliferation assay (S3 Fig). Thus, the events that initiated irreversible cell killing occurred relatively rapidly.

**Fig 3:**
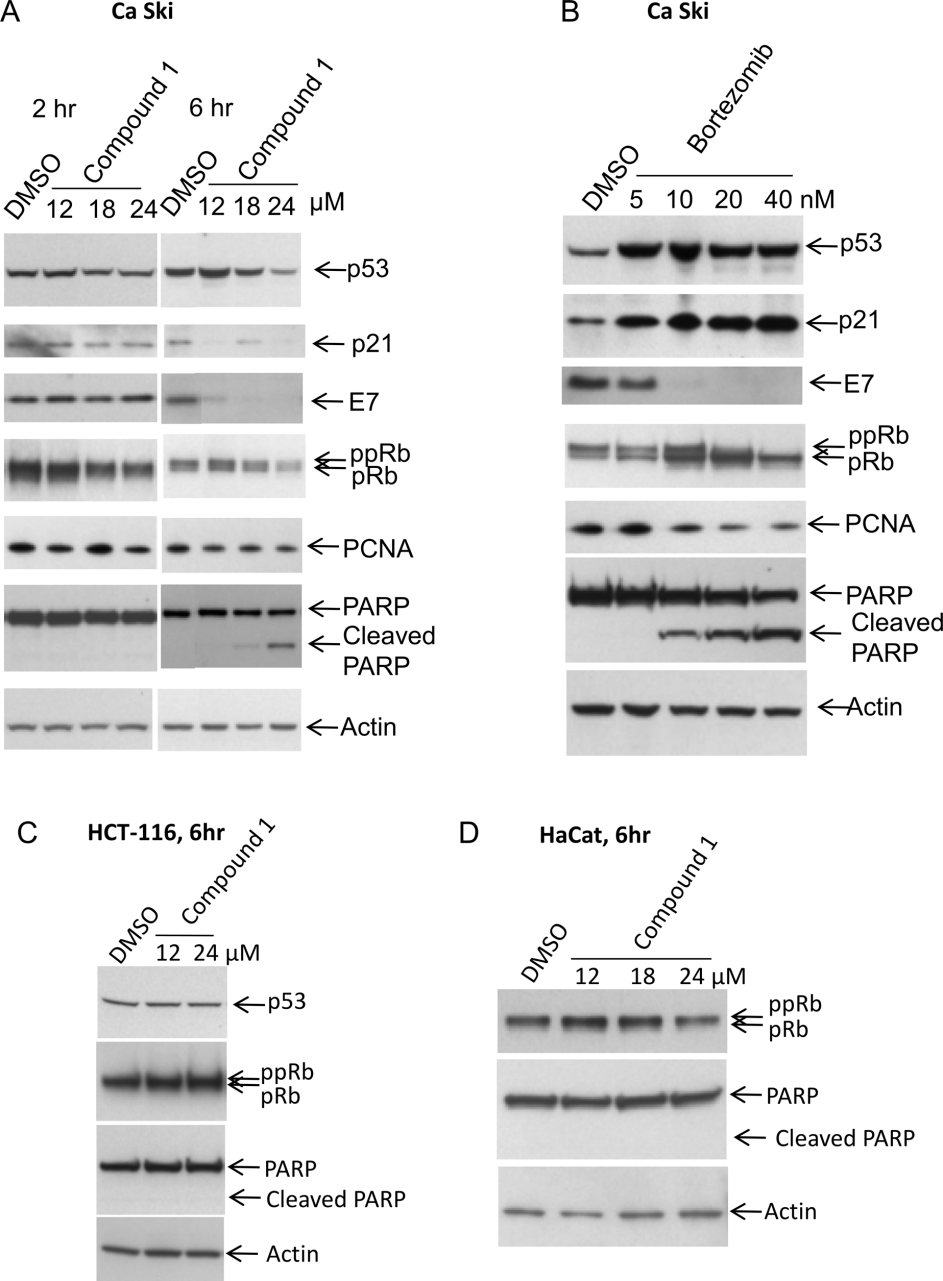
Effect of Compound 1 on protein expression in treated cells. Lysates from Ca Ski cells treated with DMSO or 12, 18 and 24 μM Compound 1 (A) or 5, 10, 20 and 40 nM bortezomib (B) were separated by SDS-PAGE and probed with antibodies against the specific proteins listed, as described in Materials and Methods. Western blots of Compound 1 treated HPV-negative cell lines HCT-116 and HaCat are shown in panels C and D.

Since both HPV E6 and E7 proteins are important for transformation by high risk HPV types, the effect of Compound 1 on changes in the expression of these proteins and their downstream targets was examined. Reduction in E6 protein in response to E6 specific RNA interference (RNAi) has been shown to stabilize p53, resulting in an increase in the overall level of p53 protein [28]. In response to stabilization of p53, an increase in the relative level of p53 target protein, p21, was also noted. Similarly, a reduction in HPV E7 levels, through treatment with RNAi, has been shown to stabilize the Rb protein and promote its hypophosphorylation [28]. To test for effects of Compound 1 on the p53 and Rb pathways, Ca Ski cells were treated with varying concentrations of Compound 1 (Fig 3A) or with the proteosome inhibitor bortezomib as a control (Fig 3B) for either 2 or 6 hours, and cell lysates were subjected to western immunoblotting with antibodies specific for viral or cellular proteins. Unfortunately, no commercially available antibodies to HPV 16 E6 showed sufficient specificity in our experiments, and in-house efforts to generate E6 antibodies were also unsuccessful (data not shown). As a surrogate for the E6 protein, the level of p53 protein present within Ca Ski cells was measured. Upon treatment with 12 μM Compound 1 (Fig 3A), there was a modest increase in p53 levels, particularly after 6 hours, when compared to DMSO treated control. However, p53 levels decreased at higher concentrations of Compound 1. It is notable that there was not a corresponding increase, but rather a decrease, in p21 levels upon treatment with compound. This is in stark contrast to the substantial increases in the levels of both p53 and p21 observed upon treatment with the proteosome inhibitor bortezomib (Fig 3B), which are consistent with its reported inhibition of the proteasomal degradation of p53 [29, 30]. The level of p53 expression did not change in Compound 1 treated HPV-negative HCT-116 cells (Fig 3C).

Using an antibody specific for HPV E7 protein we observed a significant decrease in E7 protein upon treatment with Compound 1; however, this was observed only at the later time point (Fig 3A). When the same lysates were probed with antibody that recognizes both the hypo- and hyper-phosphorylated forms of Rb, we observed a decrease in total Rb protein in cells treated with 18 or 24 μM Compound 1, but no overall change in the relative abundance of the hypo- (pRb) and hyper-phosphorylated (PPRb) forms of Rb (Fig 3A). This is in contrast to the drastic decrease in HPV E7 levels and in the accumulation of pRb in response to treatment with bortezomib (Fig 3B). The hypo-phosphorylation of Rb promotes its binding to E2F and targets E2F for degradation. Proliferating Cell Nuclear Antigen (PCNA), a target of E2F-mediated activation, was not affected by treatment with Compound 1 (Fig 3A), whereas the level of PCNA decreased with increasing concentrations of bortezomib (Fig 3B). No substantial decrease in Rb levels was observed in the HPV-negative HCT-116 or HaCat cell lines upon treatment with Compound 1 (Fig 3C and 3D). Thus, treatment with Compound 1 did not have the effects that might be expected to accompany changes in the expression of HPV E6 or E7 proteins.

In HPV-transformed cells, decreases in E7, p53, and Rb in response to Compound 1 treatment coincided with induction of Caspase-3 mediated cleavage of PARP (Fig 3A). These results were confirmed in another HPV-transformed cell line, SCHPV-18 (S2 Fig). PARP cleavage was not detected in HPV negative HaCat or HCT-116 cells treated in parallel (Fig 3C and 3D). This result suggested that cell death in response to treatment with Compound 1 occurs selectively in HPV-positive cell lines via induction of apoptosis.

**Fig 4:**
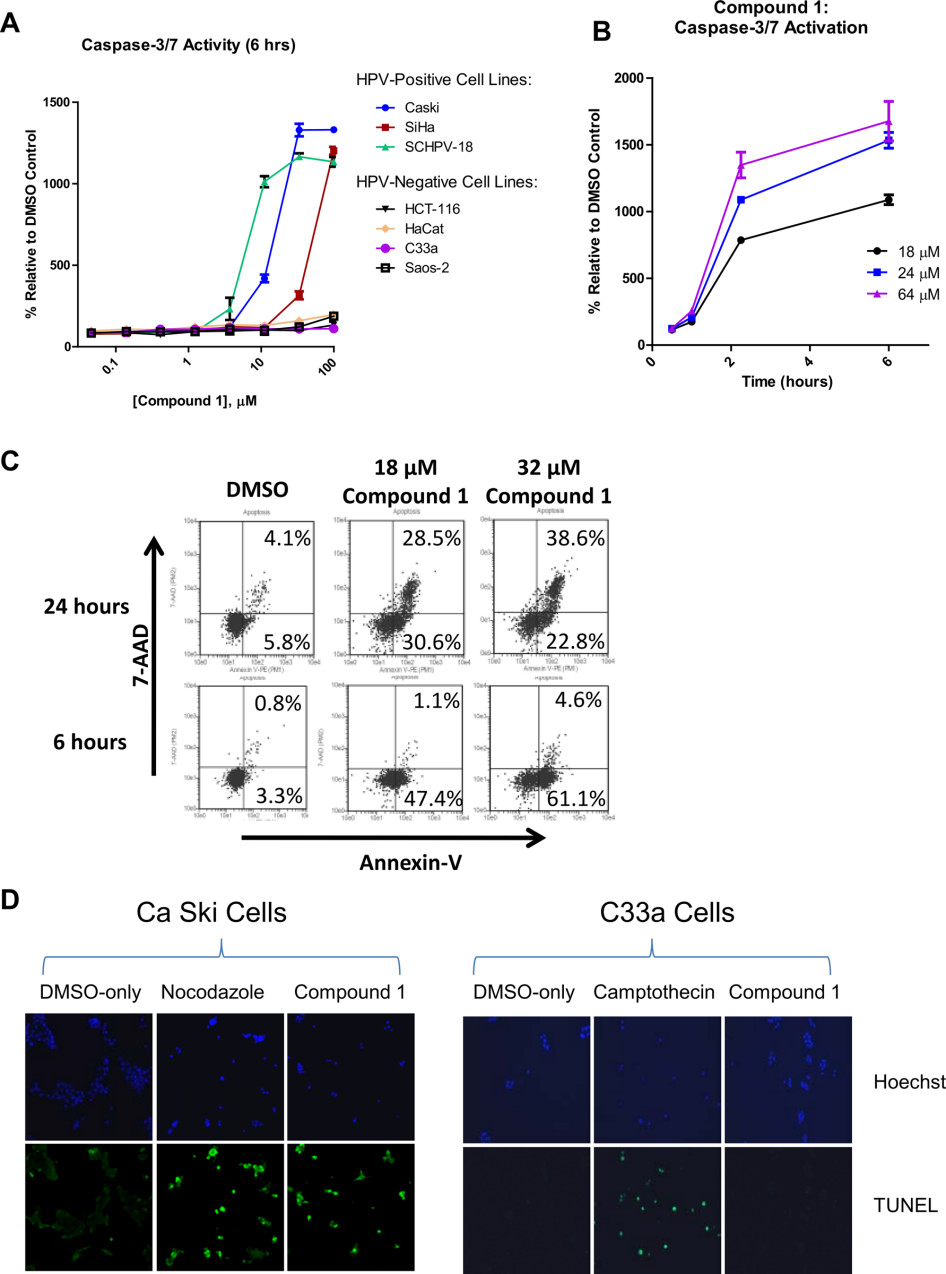
Effect of Compound 1 on markers of apoptosis in treated cells. (A) HPV-positive and HPV-negative cell lines were treated with increasing concentrations of Compound 1 for 6 hours, and then tested for Caspase-3/7 activity. (B) Ca Ski cells were treated with the indicated concentrations of Compound 1 and tested for Caspase-3/7 activity at various times. The graph shows mean values of percent Caspase-3/7 activity relative to DMSO-treated controls. Each point represents the mean of triplicates, with error bars indicating standard deviation. (C) Ca Ski cells were treated with DMSO or the indicated concentrations of Compound 1 for 6 or 24 hours, and then stained with Annexin-V and 7-AAD prior to flow cytometry. (D) HPV-positive Ca Ski or HPV-negative C33a cells treated for 24 hours with DMSO, 18 μM Compound 1, or the control inhibitors 5 μM nocodazole or 1 μM camptothecin. Cells were fixed and stained with either the DNA stain Hoechst or by TUNEL, as indicated.

In order to further define apoptotic events associated with Compound 1 treatment, cells were incubated with or without Compound 1, and then tested for the presence of effector Caspase-3/7 activity using a luciferase-based assay. Titration of Compound 1 on various HPV-positive and -negative cell lines resulted in greater than 10-fold activation of the caspases in HPV-transformed cell lines Ca Ski, SiHa, and SCHPV-18 relative to DMSO-treated control cells (Fig 4A). In contrast, the HPV-negative cell lines Saos-2, C33a, HaCat, and HCT-116 showed minimal (≤ 2-fold) activation of caspase activity even at the highest concentration of Compound 1 tested (100 μM). These results demonstrate a selective induction of caspases in HPV-transformed cells relative to HPV-negative cells. In a time course of caspase induction in Ca Ski cells, treatment with three concentrations of Compound 1 resulted in greater than 7-fold induction of caspase activity, as early as 2 hours post-treatment, indicating that apoptosis was initiated relatively early after compound addition. This level of induction was maintained at 6 hours post-treatment. These results suggest that activation of Caspase-3/7 may precede the reduction in E7 levels observed at 6 hours in Fig 3A. Quantitation of E7 protein in Caski cells by high content microscopy confirmed little if any effect at 2 hours post-treatment. The reduction in E7 protein peaked at later time points, 6 and 24 hours post-treatment (S4 Fig).

To confirm the progression through apoptosis, HPV-positive Ca Ski cells were stained with Annexin-V and 7-AAD (Fig 4C). As cells were exposed to Compound 1 for either 6 or 24 hours, they progressed from predominantly Annexin-V positive/7-AAD negative at 6 hours, indicative of early apoptosis, to Annexin-V positive/7-AAD positive at 24 hours post-treatment, indicative of late stage apoptosis. Ca Ski and C33a cells were also tested for the presence of double-stranded DNA (dsDNA) breaks, characteristic of chromosome fragmentation that occurs during the late stages of apoptosis, using a TUNEL (Terminal deoxynucleotidyl transferase dUTP Nick End Labeling) assay (Fig 4D). Bright staining indicative of the presence of dsDNA breaks was apparent in Ca Ski cells treated with Compound 1, as well as in cells treated with nocodazole, an inhibitor of mitosis known to induce dsDNA [31, 32]. In addition, counter-staining with Hoechst confirmed the presence of bright, picnotic nuclear staining and blebbing known to occur during the late stages of apoptosis. Treatment of HPV-negative C33a cells with Compound 1 under conditions identical to those used for HPV-positive cells did not result in staining in the TUNEL assay (Fig 4D). Although the same concentration of nocodazole used to treat Ca Ski cells did not induce TUNEL staining in C33a cells (data not shown), C33a cells could be induced to undergo apoptosis by treatment with camptothecin, a known inducer of intrinsic apoptosis through inhibition of dsDNA break repair [33] (Fig 4D). Taken together, the results shown in Fig 4 demonstrate that Compound 1 selectively induces events characteristic of apoptosis in HPV-transformed cells.

**Fig 5:**
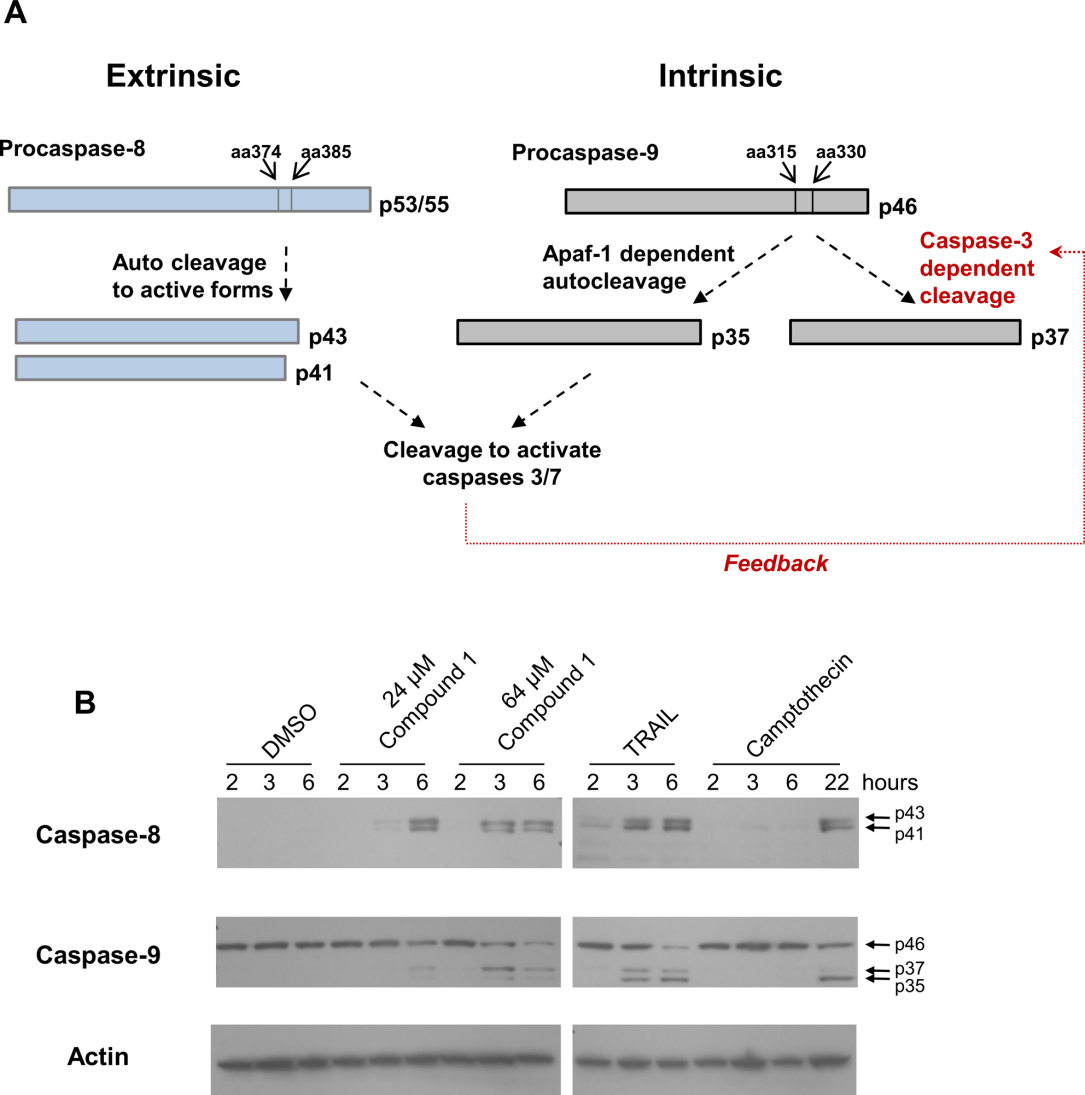
Effects of Compound 1 on activation of Caspases 8 and 9. (A) The diagram depicts the full-length Procaspase-8 and Procaspase-9 proteins and their cleaved products formed upon activation (adapted from[35] and [36]). (B) Ca Ski cells were treated with DMSO, Compound 1 (24 or 64 μM), 0.1 μg/mL TRAIL or 5 μM camptothecin for the indicated times. Western blots of lysates from treated cells were probed with antibodies directed against the Caspase-8 cleavage products, p41 and p43 (top panel), Procaspase-9 and its cleaved forms, p35 and p37 (middle panel) or Actin (bottom panel) as a control for sample loading.

Apoptosis can be induced through either extrinsic or intrinsic pathways and the two pathways activate distinct initiator caspases, either Caspases-8/10 or Caspase-9, respectively. To determine which of these pathways is induced by Compound 1, immunoblotting was performed with antibodies that detect Caspase-8 or -9 proteins (Fig 5). The expected sizes of various Caspase-8 and -9 activated cleavage products and the relative order of cleavage events are diagrammed in Fig 5A. Using an antibody specific for the p41 and p43 cleaved forms of Caspase-8, treatment with Compound 1 resulted in the detection of both cleavage products at 3 and 6 hours post-treatment (Fig 5B). The active forms of Caspase-8 were not detected in DMSO-treated control samples. When Ca Ski cells were treated with TRAIL, which induces apoptosis through binding to death receptors 4 and 5 (DR 4/5), the p41/p43 cleaved forms of Caspase-8 were detected at 2, 3, and 6 hours post-treatment. Treatment of Ca Ski cells with Camptothecin also resulted in detectable Caspase-8 cleavage, but only at 22 hours post-treatment. Caspase-8 activation by Camptothecin is likely a result of cross-talk between the intrinsic and extrinsic pathways, since induction of intrinsic apoptosis can up-regulate p53-dependent expression of death receptors and ligands[34]. Detection of the activated forms of Caspase-8 by Compound 1 suggests that this compound activates the extrinsic apoptotic pathway.

Western immunoblots of the samples were also probed with an antibody that recognizes both the inactive full length and the cleaved and activated forms of Caspase-9. Apaf-1 co-factor-dependent autocleavage and Caspase-3-dependent feedback activation of Caspase-9 result in two distinct cleavage products p35 and p37, respectively (Fig 5A), both of which are indicators for activation of intrinsic apoptosis (reviewed in [36]). When Ca Ski cells were treated with camptothecin, only the 35 kDa band was detected after 22 hours of treatment, consistent with intrinsic apoptosis. In contrast, treatment with TRAIL for 3 hours resulted in both the p35 and p37 forms of Caspase-9. In TRAIL-treated cells, the p37 form can be generated by feedback cleavage of Caspase-9 by Caspase-3. The p35 form of Caspase-9 generated upon TRAIL treatment may result from cross-talk between the extrinsic and intrinsic pathways which can occur through Caspase-8 cleavage of BID, its translocation to the mitochondria, and activation of Apaf-1. Upon treatment with TRAIL, the amount of p35 relative to p37 increased from 3 to 6 hours, consistent with prior activation of Caspase-3. In contrast, p37 was the predominant form observed at 6 hours in cells treated with 24 μM Compound 1, and at 3 or 6 hours in cells treated with 64 μM Compound 1. Re-probing these samples with an antibody specific for the p37 form of Caspase-9 confirmed the identity of this band (data not shown). Caspase-9 cleavage to p37 in response to treatment with Compound 1 likely occurs through feedback by Caspase-3/7 (Fig 5A). The presence of predominantly the p37 Caspase-9 cleavage product and the concomitant activation of Caspase-8 together suggest that Compound 1 initially triggers apoptosis through the extrinsic pathway and subsequently engages the intrinsic pathway through feedback cleavage of Caspase-9 by Caspase-3/-7.

## Discussion

Results presented here demonstrate that a quinoxaline compound, Compound 1, selectively inhibits the proliferation of HPV-transformed cells. Inhibition of proliferation did not directly correlate with the well-characterized functions of HPV E6 and E7 proteins in modulation of the p53 and Rb pathways. Instead, the anti-proliferative effects of Compound 1 are consistent with induction of extrinsic apoptosis.

We observed a marked down-regulation of HPV-16 E7 protein in Ca Ski cells following treatment with Compound 1, but the timing of the observed reductions in E7, as well as in the levels of p53, and Rb proteins, coincided with maximal induction of Caspase-3/7 activity. During apoptosis, specific intra- or extracellular stimuli initiate a cascade of caspase-dependent events that ultimately result in cessation of protein synthesis, fragmentation of cellular DNA, and membrane blebbing. Down-regulation of protein synthesis in apoptosis is caused by caspase-dependent cleavage of eukaryotic translation initiation factors eIF2α, eIF4B, and eIF4G (reviewed in [11]). Since the half-life of HPV E7 transcript and protein is relatively short, a change in protein level due to the decrease in protein synthesis may be more apparent at earlier time points than with longer-lived gene products [12]. Since reduced expression of E7 in Compound 1 treated cells does not occur early in the apoptotic process, but rather coincides with activation of the caspases responsible for turning off protein synthesis, this suggests that the reduction of E7 protein is a result of apoptotic events rather than the cause of apoptosis.

Apoptosis can be induced through extrinsic, death receptor-mediated, or intrinsic, mitochondria-mediated pathways. The two pathways activate distinct initiator caspases, Caspases-8/10 or Caspase-9, respectively, and converge at a later stage when downstream effector caspases, including Caspases-3 and -7, are activated. Extrinsic apoptosis is initiated by the binding of ligands, such as Fas Ligand, TRAIL, and TNFα to their cognate cell surface death receptors Fas Receptor, Death Receptors 4 and 5 (DR 4/5), and TNF-Receptor 1 (TNF-R1), respectively (reviewed in [37]). Upon ligand binding, a multi-protein signaling complex termed the DISC (death inducing signaling complex) is assembled on the intracellular side of the plasma membrane to initiate downstream signaling events. Two components of the DISC, FADD and TRADD, are adaptor proteins that serve to recruit Procaspases-8 and -10 to the DISC. Receptor binding and subsequent caspase dimerization activate these caspases, which in turn cleave effector Caspases-3 and -7 to carry out the downstream events of apoptosis. The identification of activated forms of Caspase-8 at early time points post-treatment strongly suggests that Compound 1 activates apoptosis through the extrinsic pathway.

While detectable cleavage of Caspase-9 was observed in cells treated with Compound 1, several lines of evidence suggest apoptosis is not initiated intrinsically but instead is a downstream event resulting from Caspase-8 cleavage. The intrinsic pathway is initiated through intracellular events, such as DNA damage-induced activation of p53-dependent PUMA expression (reviewed in [34]). These events perturb the mitochondria, resulting in release of cytochrome C, which then initiates formation of the apoptosome complex, containing procaspase-9 and Apaf-1. A conformational change in this complex results in auto-cleavage of Caspase-9 to its active form. We did not observe an increase in expression of the p53 targets p21 or PUMA, or a decrease in the anti-apoptotic factor Bcl-2 (S2 Fig) upon Compound 1 treatment, as might be expected for induction of intrinsic apoptosis. After activation, cross-talk between the extrinsic and intrinsic pathway pathways can occur in some cell types, resulting in an amplification of apoptotic effects. Treatment with Compound 1 resulted in predominantly the p37 form of Caspase-9, which is associated with indirect activation through feedback cleavage of Procaspase-9 by Caspases-3/7 rather than direct activation through the mitochondrial pathway. When the extrinsic pathway is activated, Caspase-8 cleavage of BID can trigger cytochrome C release and subsequent events in the intrinsic apoptotic pathway. Upon treatment with TRAIL, an activator of extrinsic apoptosis, we observed BID cleavage, accounting for the presence of the p35 form of Caspase-9 in these cells (S2 Fig). We observed a very low level of the p35 cleavage product of Caspase-9 in Compound 1 treated cells consistent with the potential for Caspase-8 activation of BID. Despite the possibility of this feedback loop being active in Compound 1 treated cells, BID cleavage was not detected with this compound (S2 Fig). Whether this is due to a difference in the overall strength of activation of Caspase-8 between the two treatments, or due to a real difference between their mechanisms of action remains unclear.

It is unclear whether the transformed state of HPV-positive cells renders them less susceptible to apoptosis compared to HPV-negative cells. Many of the cell lines used here are known to express at least some of the death receptors, ligands and adaptor proteins, but HPV transformation may prevent downstream functions. For example, E6 has been implicated in protecting cells from TNF-mediated apoptosis through binding the TNF receptor and preventing its association with the adaptor protein TRADD [38, 39]. Conversely, HPV-negative cells may be intrinsically less susceptible to extrinsic apoptosis if they lack some signaling pathways. It is possible that some cell lines may lose their ability to respond to apoptotic signals as a consequence of multiple passages through maintenance of the cell lines. Interestingly, a new stably transformed cell line, SCHPV-18, established directly from primary keratinocytes through transfection of the HPV-18 genome (S1 Fig) was susceptible to the effects of Compound 1 (Fig 2A). This result suggests that transformation with high risk HPV genomes may render the cells susceptible to this compound. Unfortunately, we were unable to assess the effect of this compound on non-transformed primary keratinocytes since in our hands these cells were not readily maintained in a healthy state in the absence of HPV transformation in culture.

Several lines of evidence suggest that HPV actively inhibits the extrinsic pathway in transformed cells, presenting a potential explanation for the selectivity of Compound 1 for HPV-transformed cells. In isolates from HPV-positive cervical neoplasias, expression of both death receptors and their ligands has been detected in the absence of apoptosis, suggesting the extrinsic apoptosis pathway has somehow been disrupted [40]. The HPV E6 protein has been shown to bind the adaptor protein FADD as well as Procaspase-8, mediating their degradation to protect cells from TRAIL-mediated apoptosis [41–44]. In fact, a recent report identified flavonol compounds capable of inhibiting the *in vitro* interaction of HPV E6 with Caspase-8[45]. However, introduction of a higher level of exogenous E6 or E7 into Ca Ski cells using recombinant lentiviruses [46] failed to rescue cells from Compound 1-induced cell death (S1 Table). Transduction of mouse J2-3T3 cells with recombinant lentiviruses expressing E6 or E7 also failed to sensitize the cells to Compound 1 (data not shown). While this could indicate that a non-conserved cellular component is missing in mouse cells, alternatively it may suggest that the E6 and E7 proteins do not directly participate in the effects of the compound. Since the entire HPV genome is present in the HPV transformed cells used in these studies, a role for another HPV protein in extrinsic apoptosis cannot be ruled out. Along those lines, studies in HaCat cells have implicated the HPV E5 protein in down-regulation of Fas- and TRAIL-mediated apoptosis [47, 48].

At present it is unclear how Compound 1 might induce apoptosis in HPV-transformed cells. One potential mechanism involves direct binding of the compound to the extracellular death receptor to cause its activation, but it is just as likely that the compound might affect an early intracellular signaling event. For example, the compound could directly activate Procaspase-8, or disrupt binding of inhibitory cellular proteins, cFLIPs (cellular FLICE inhibitory proteins), which block Procaspase-8 activation [49], or inhibit the activity of cIAP (cellular inhibitor of apoptosis) proteins that serve to inhibit Caspase-8 dependent apoptosis in response to TNF-α signaling [50]. More studies will be required to fully understand the mechanism of action of Compound 1. However, we favor a model where the compound acts by directly affecting the extracellular components of the death receptor, since binding of biotin-labeled compound to fixed cells can be detected using labeled neutravidin, a protein too large to diffuse into non-permeabilized cells (data not shown).

Through medicinal chemistry efforts, a series of related compounds with selective activity were identified, and the difluoromethoxy moiety was found to be necessary for anti-proliferative effects on HPV-positive cells (data not shown). Unfortunately, this series of inhibitors was not pursued further because the difluoromethoxy moiety was susceptible to chemical reactivity with cysteine and other amino acid side chains (data not shown). While this reactivity is a potential liability to further development, it could be exploited to label and identify interacting proteins and thus identify the molecular target of Compound 1, a first step toward finding a more desirable, chemically non-reactive chemotype. The discovery of this compound series shows a proof of concept that high risk HPV transformed cells can be selectively induced to undergo drug-mediated apoptosis. It would be of clinical interest if a chemically tractable series with this biological effect could be identified and advanced through drug development.

## Supporting Information

S1 Fig Detection of integrated HPV DNA in the SCHPV-18 and SCHPV-31 cell lines. (A) Southern blot on 10 μg of total cellular DNA from HPV-negative cell line C33a (lane 1) or the HPV-transformed cell lines HeLa and SCHPV-18 (lanes 3 and 4) or (B) C33a (lane 1), 9E/HPV31 cells containing the episomal HPV31 genome or SCHPV-31 cells (lanes 3 and 4). Blots were probed with radiolabeled HPV18 (A) or HPV-31 (B) genome. Lane 2 in each panel contained no DNA.
(TIF)

S2 Fig Effect of Compound 1 on protein expression in treated cells. Western immunoblots of lysates from SCHPV-18 cells (A) or Ca Ski cells (B) using the indicated antibodies. Samples were prepared by treatment of cells in the presence of DMSO, various concentration of Compound 1 or 0.1 μg/mL TRAIL for the indicated times.
(TIF)

S3 Fig Effects of the length of Compound 1 treatment on anti-proliferative activity in Ca Ski cells in a 4 day assay. Medium containing Compound 1 was added on day 0. At the indicated time points, compound-containing medium was removed, cells were gently washed with DPBS twice, and replaced with medium lacking Compound 1.
(TIF)

S4 Fig High-content HPV oncoprotein E7 quantification assay. HPV positive Ca ski cells were incubated in the presence of 24 μM Compound 1 for the indicated times. E7 protein was detected and quantitated by high content microscopy using an antibody specific for HPV E7.
(TIF)

S1 Table Activity of Compound 1 in Ca Ski cells transduced with lentiviruses expressing HPV E6 and/or E7. (TIF)

## Abbreviations

HPV: Human Papilloma Virus
IgG: Immunoglobulin
TUNEL: Terminal deoxynucleotidyl transferase dUTP nick end labeling
PARP: poly(ADP-ribose) polymerase
