# Supplementary figures and images for: A Small Molecule Inhibitor Selectively Induces Apoptosis in Cells Transformed by High Risk Human Papilloma Viruses
Source: PLoS One. 2016 Jun 9;11(6):e0155909. doi: 10.1371/journal.pone.0155909 (PMC4900674; doi:10.1371/journal.pone.0155909)

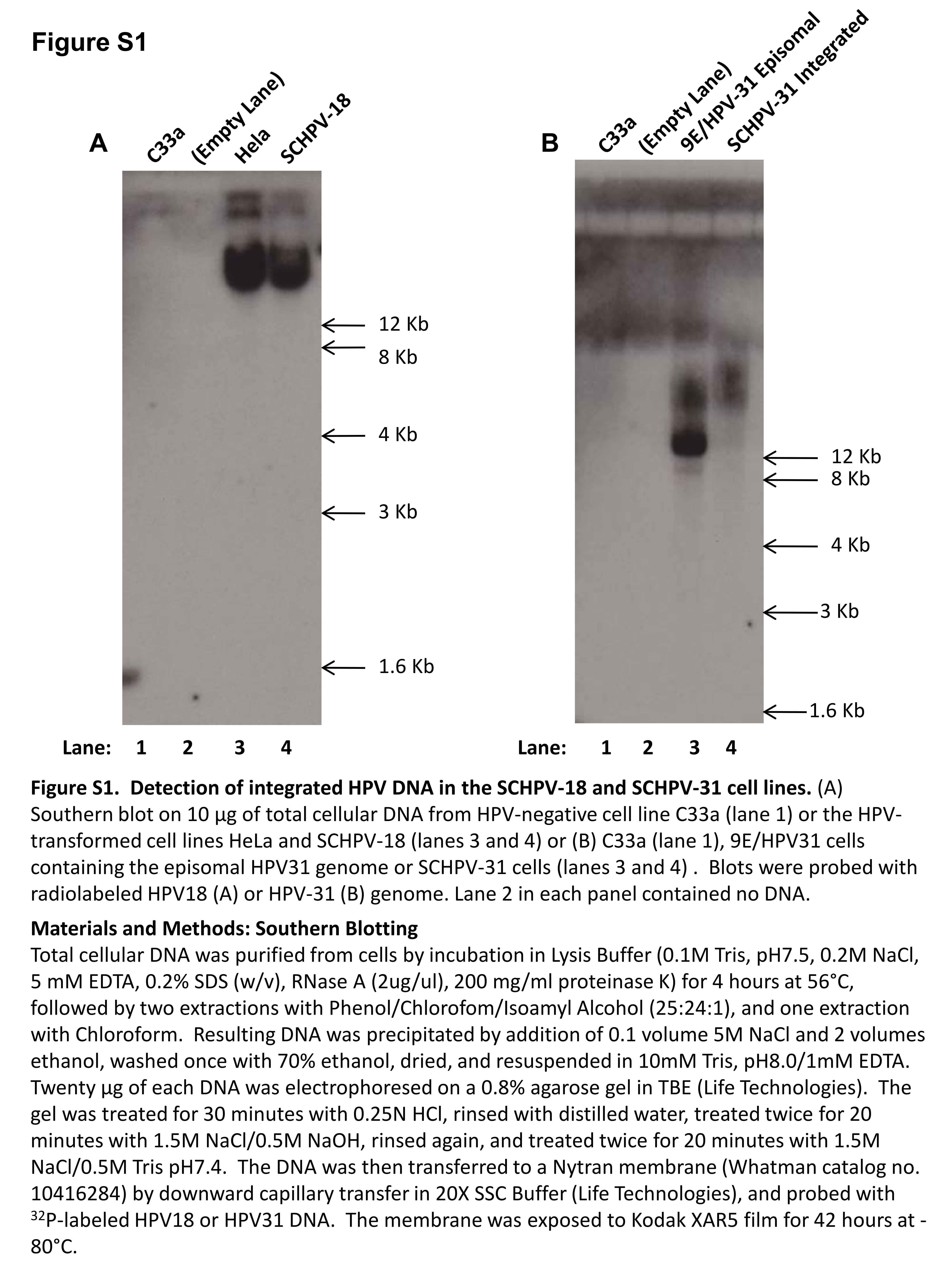

Supplement: S1 Fig — (A) Southern blot on 10 μg of total cellular DNA from HPV-negative cell line C33a (lane 1) or the HPV-transformed cell lines HeLa and SCHPV-18 (lanes 3 and 4) or (B) C33a (lane 1), 9E/HPV31 cells containing the episomal HPV31 genome or SCHPV-31 cells (lanes 3 and 4). Blots were probed with radiolabeled HPV18 (A) or HPV-31 (B) genome. Lane 2 in each panel contained no DNA. (TIF) [file pone.0155909.s001.tif]

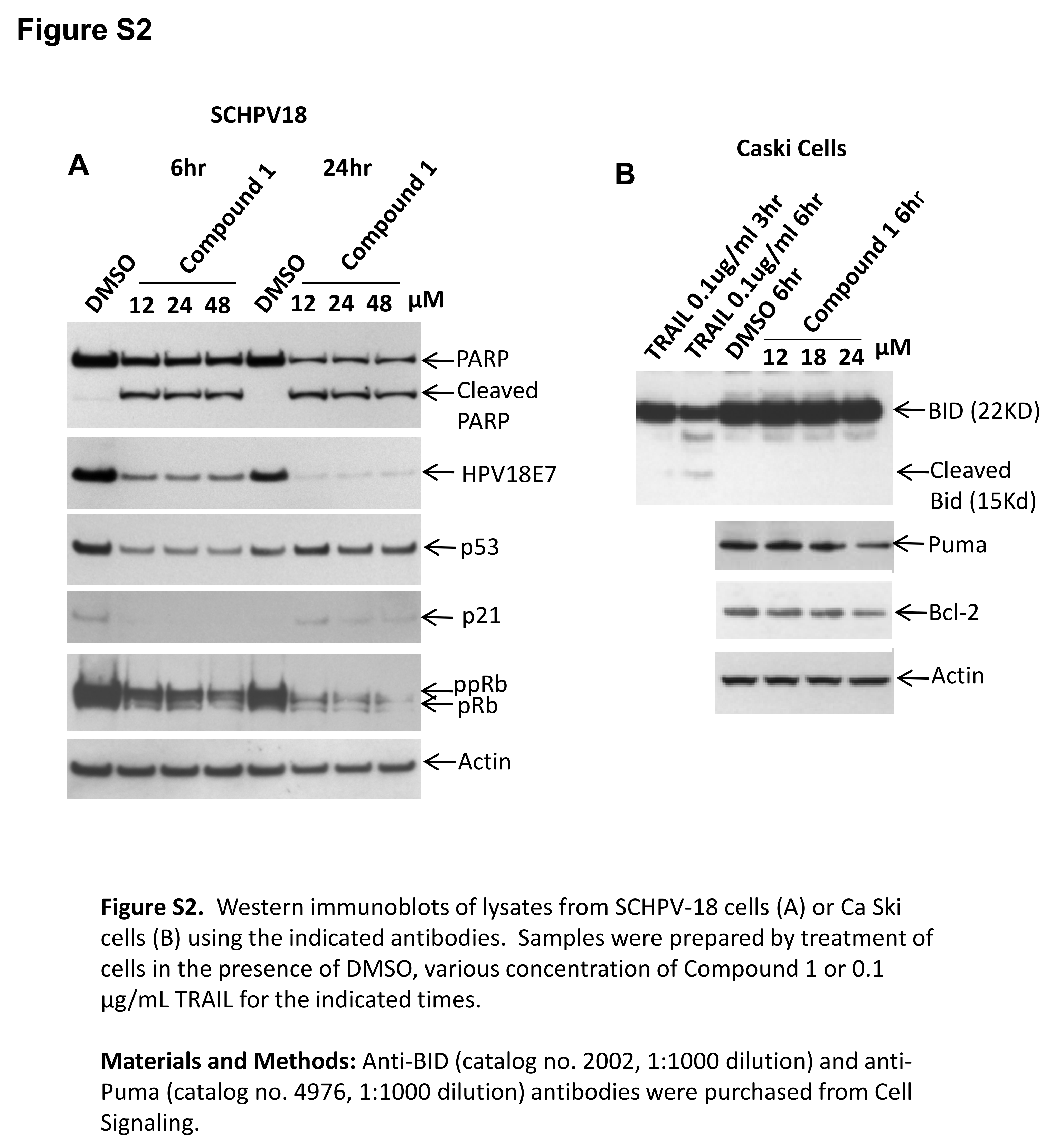

Supplement: S2 Fig — Western immunoblots of lysates from SCHPV-18 cells (A) or Ca Ski cells (B) using the indicated antibodies. Samples were prepared by treatment of cells in the presence of DMSO, various concentration of Compound 1 or 0.1 μg/mL TRAIL for the indicated times. (TIF) [file pone.0155909.s002.tif]

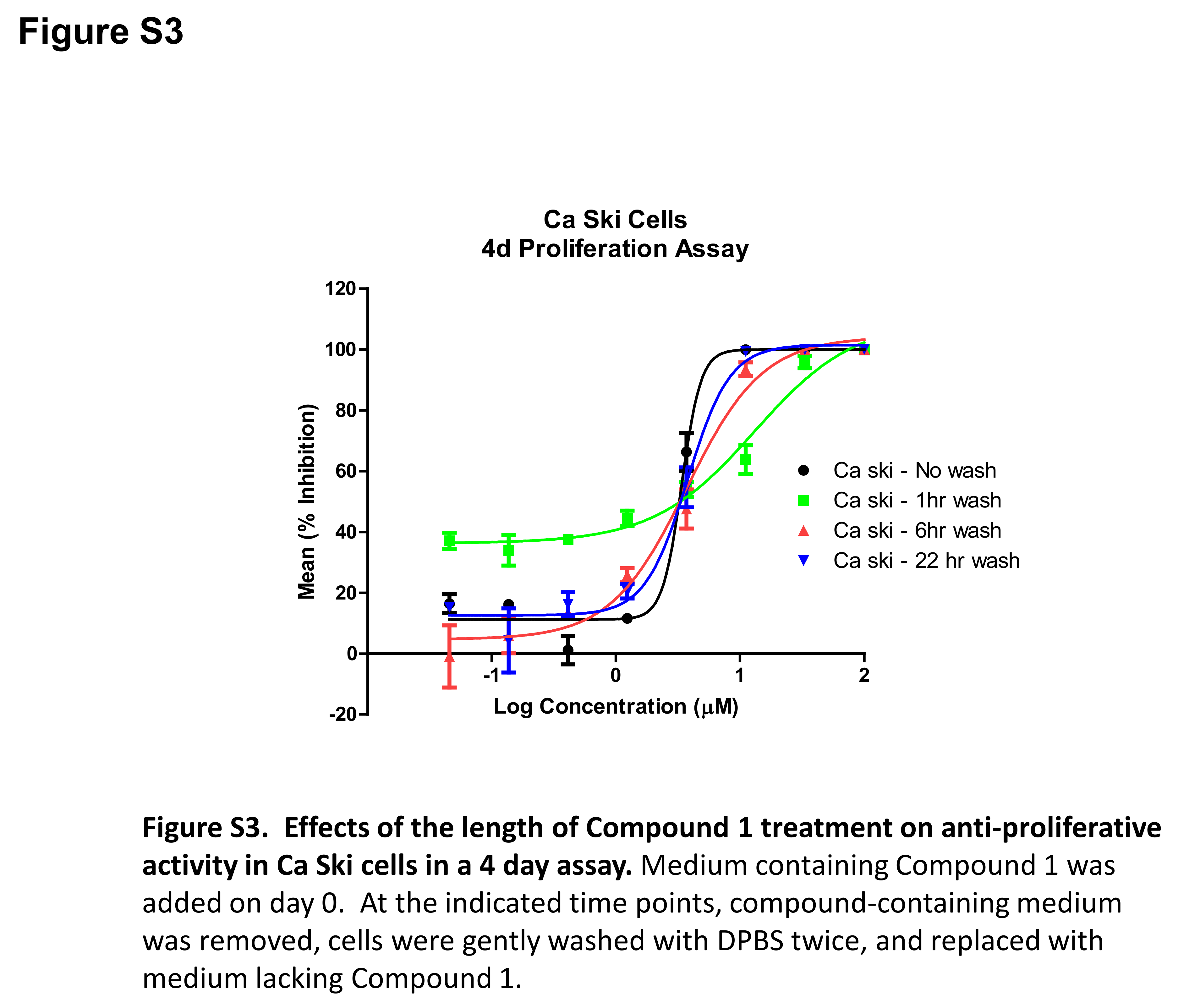

Supplement: S3 Fig — Medium containing Compound 1 was added on day 0. At the indicated time points, compound-containing medium was removed, cells were gently washed with DPBS twice, and replaced with medium lacking Compound 1. (TIF) [file pone.0155909.s003.tif]

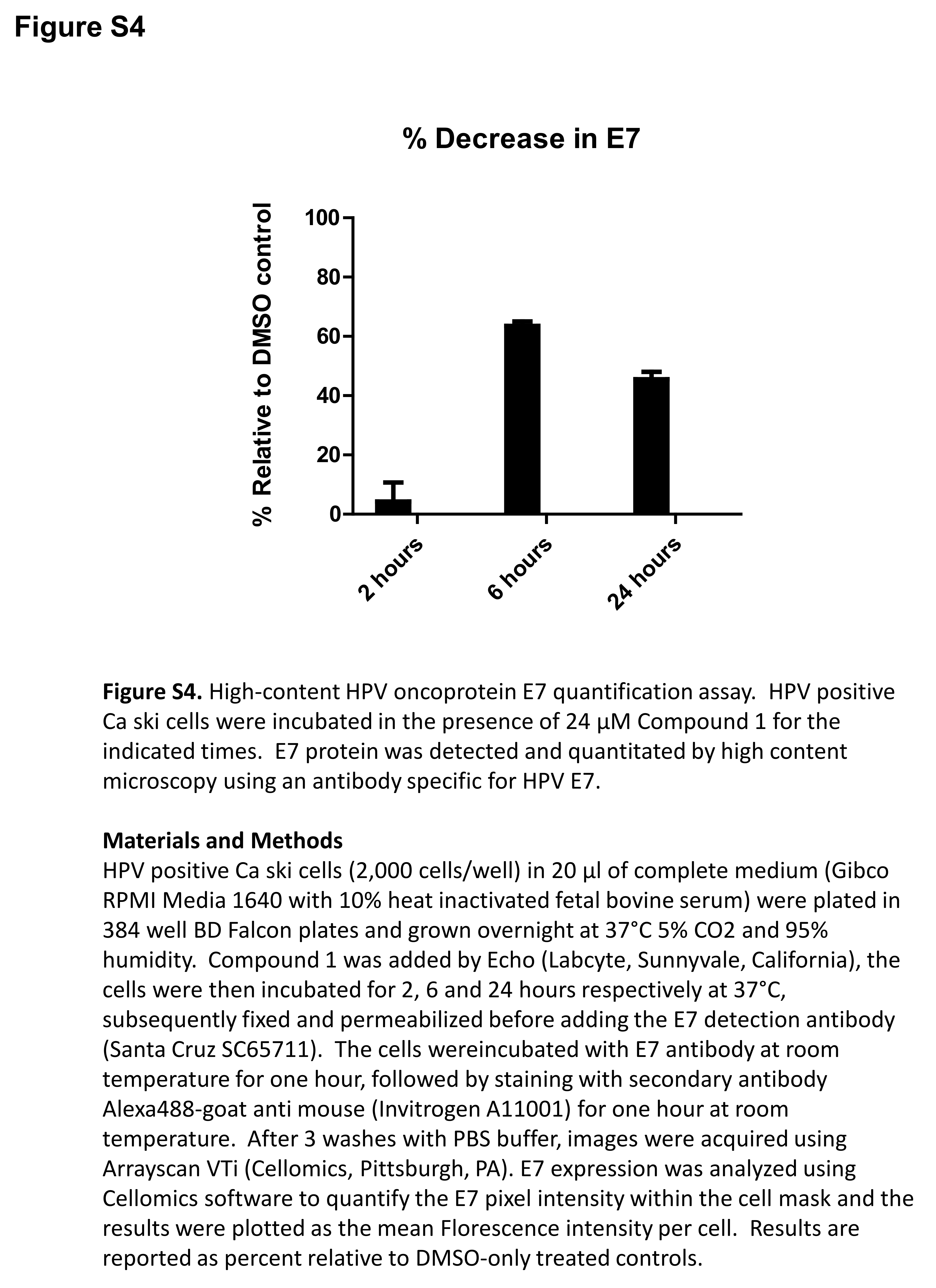

Supplement: S4 Fig — HPV positive Ca ski cells were incubated in the presence of 24 μM Compound 1 for the indicated times. E7 protein was detected and quantitated by high content microscopy using an antibody specific for HPV E7. (TIF) [file pone.0155909.s004.tif]

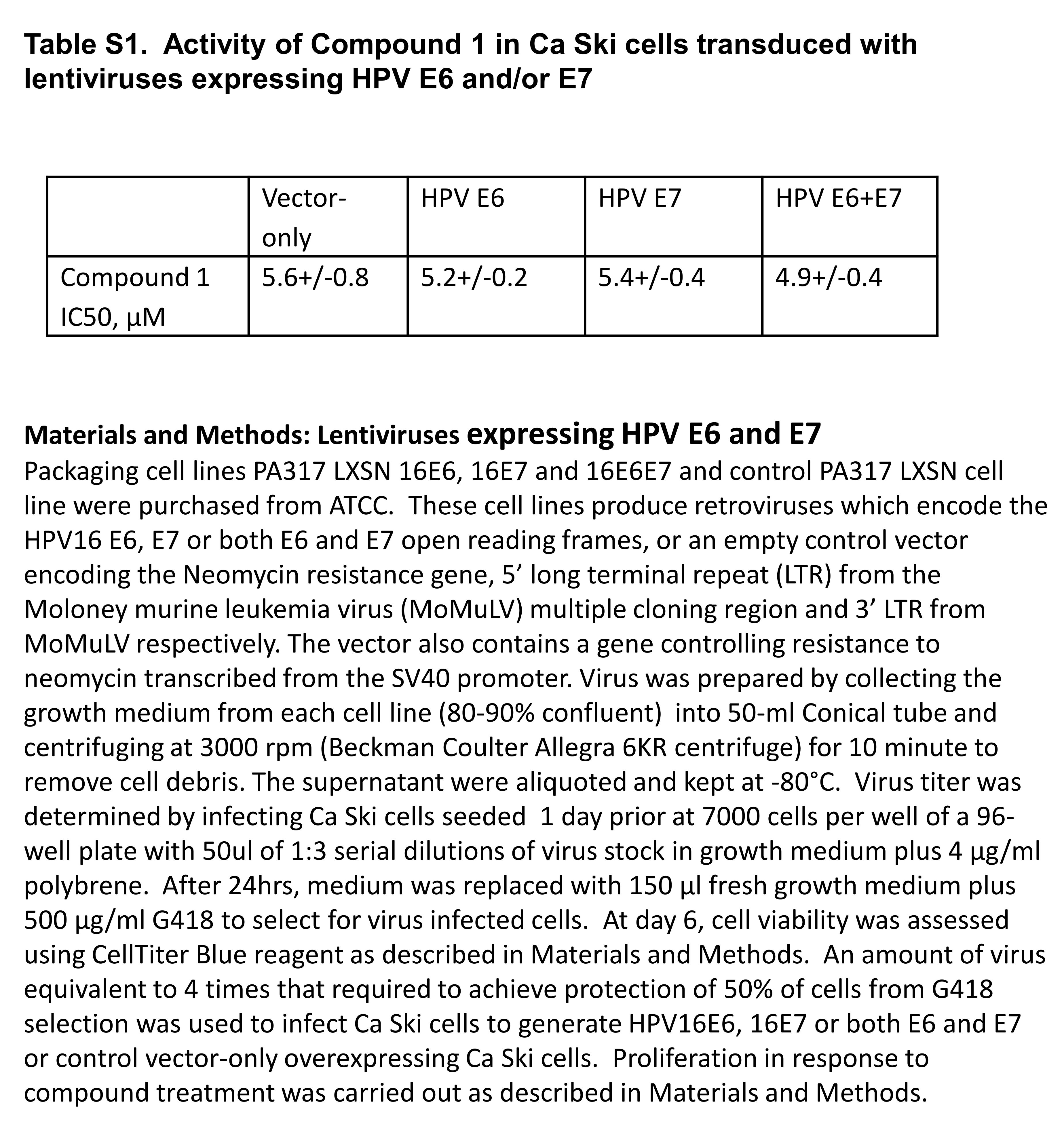

Supplement: S1 Table — (TIF) [file pone.0155909.s005.tif]
